# The Effect of Cigarette Smoking on Human Sperm Creatine Kinase Activity: As An ATP Buffering System in Sperm

**Published:** 2013-03-03

**Authors:** Mohammad Ali Ghaffari, Morad Rostami

**Affiliations:** Department of Biochemistry, Cellular and Molecular Research Center, Physiology Research Center, School of Medicine, Ahwaz Jundishapour University of Medical Sciences, Ahwaz, Iran

**Keywords:** Cigarette Smoking, ,, Sperm, Creatine Kinase

## Abstract

**Background:**

Spermatozoa are a group of cells that consume adenosine triphosphate (ATP) rapidly. Creatine kinase (CK), produced by creatine phosphate, is an energy reservoir for the rapid buffering and regeneration of ATP and can play an important role in sperm motility. Therefore, this study investigates the effects of cigarette smoking on human sperm CK activity in males who smoke.

**Materials and Methods:**

In this case - control study, we obtained semen samples from male smokers (n=64) and nonsmokers (n=83). Smokers were categorized as light, moderate, or heavy smokers according to the daily number of cigarettes smoked and the number of years they have smoked. Data were analyzed by the independent t test and Pearson’s analysis.

**Results:**

This investigation showed significantly lower sperm CK activity and movement in male smokers compared to nonsmokers. In addition, it was demonstrated that cigarette smoking had a dose-dependent effect on these parameters. There was a positive relation, although not significant, between sperm CK activity and its motility in male smokers.

**Conclusion:**

Smoking, by diminishing sperm CK activity, may potentially impair sperm energy homeostasis and have an association with damage to sperm motility. This effect can be an important mechanism that may cause infertility in male smokers. However, further research is necessary to elucidate the underlying mechanism of sperm motility damage caused by cigarette smoking.

## Introduction

Cigarette smoking is a widely recognized health hazard and a major case of mortality. Previous studies have shown that one-third of the world’s populations over the age of 15 years are smokers (1, 2). The highest prevalence of smokers is observed in young adult males, which occurs during their reproductive years (2). Cigarette smoke contains a large number of substances including nicotine, carbon monoxide, heavy metals, benzopyrene, dimethylbenzanthracene, dimethylnitrosamine, naphthalene, and metanaphtalene (3). Inhalation of cigarette smoke leads to absorption of these substances throughout the body. It is also possible that these substances can end up in the seminal plasma of smokers via various modes of diffusion and active transport (4). Therefore, it is not surprising that cigarette smoking has a negative impact on the male reproductive system. Studies have shown that cigarette smoking affects semen quality, particularly among heavy smokers or those who have smoked for many years (2, 5)

Creatine kinase (CK) is an enzyme (EC 2.7.3.2) expressed by various tissues and cell types that require high energy. This enzyme reversibly catalyzes conversion of creatine and adenosine triphosphate (ATP) to phosphocreatine and adenosine diphosphate (ADP). Its biological role is to provide an ATP buffering system for tissues that require large amounts of energy (6). Studies show that ATP and the phosphoryl creatine shuttle are important energy sources for sperm (7, 8). Therefore, we propose that CK has an important role in sperm movement.

Numerous researches have been undertaken regarding the effect of cigarette smoking on male reproductive function (2, 5, 9-12); however, the literature that discusses this effect on human sperm CK activity as an ATP buffering system is limited. We have previously shown which nicotine, cotenine and cadmium can inhibit human sperm CK activity in an *in vitro* model (13). Therefore, the aim of this study is to investigate the relationship between cigarette smoking and sperm CK activity in male smokers.

## Materials and Methods

### Materials

ADP, adenosine monophosphate (AMP), nicotinamide adenine dinucleotide phosphate (NADP), glucose 6-phosphate dehydrogenase, hexokinase and Triton x-100 were purchased from Sigma Chemical Co. (St. Louis, MO, USA). The highest grade and purity of reagents were used in this research.

### Study population

This was a case-control study that performed in Ahwaz, Iran. Study population was selected from males who attended Razi Laboratory in Ahwaz, Iran for routine semen analyses. Prior to the collection of semen samples, information was obtained from subjects regarding their ages, occupation, smoking habits, alcohol consumption, and use of other substances and drugs. Exclusion criteria included alcohol use in the three months before study entry, recent fever, exposure to gonad toxins and heavy metals. A total of 147 men, ages 17 to 41 years were included in the study. There were 64 smokers (20-39 years) and 83 nonsmokers (17-41 years) enrolled. This study was conducted in 2009. According to the number of cigarettes smoked per day and duration of cigarettes smoked in a year, we categorized participants as either light, moderate or heavy smokers (Table 1) and short- and longterm smokers (Table 2) (5).

**Table 1:** Smoking status of study participants


Smoking status	Number of cigarettes/day	Number of cases (n)

**Non-smokers**		83
**Smokers**		64
**Light**	1-10	26
**Moderate**	11-20	30
**Heavy**	21-40	8


**Table 2:** Smoking duration of study participants


Smoking status	Duration of smoking (Y)	Number of cases (n)

**Smokers**		64
**Short-term**	1-10	43
**Long-term**	11-20	21


### Semen samples and analysis

Semen samples were collected by masturbation into sterile containers after sexual abstinence for 2 to 3 days. Semen samples were kept at 37°C and processed immediately after complete liquefaction. All semen samples were analyzed for appearance, volume, pH, sperm motility, sperm count, and sperm morphology according to World Health Organization guidelines (1). Written informed consent was obtained from study participants and the study protocol was approved by the institutional review board of the Ahwaz Jundishapour University of Medical Sciences.

### Creatine kinase (CK) isolation from sperm cells

CK of each semen sample was partially isolated as previously described by Miyaji et al. (7). Briefly, fresh semen samples were liquefied, then washed in at least 10 volumes of an ice cold solution that consisted of 30 mM tris-HCl, 80 mM NaCl, 40 mM KCl, and 0.1 mM CaCl_2_ at pH=8.2 (Merck, Darmstedt). Samples were then centrifuged at 5000 g (Centrifuge Centurion, Model K280R, UK) for 20 minutes. The resulting pellet was suspended in ice cold solution that consisted of 50 mM sodium phosphate, 150 mM NaCl, 0.2 mM EDTA, 1 mM sodium azide (Merck, Darmstedt), and 1% Triton x-100 at pH=7.2. The suspension was again centrifuged at 20000 g for 30 minutes. Afterwards, the supernatant which essentially contained all the CK was collected.

### Determination of creatine kinase (CK) activity

CK activity within seminal plasma, sperm cells and total semen of each sample were separately measured by the Rosalki method (14). This method is based upon reduction of NADP in the presence of CK, glucose, hexokinase and glucose 6-phosphate dehydrogenase. The increase in optical density (OD) at 340 nm which depends on NADP reduction is spectrophotometrically determined and provides a measure of CK activity. According to this method, one international unit (IU) of CK is the amount of enzyme which will utilize 1 µmol of creatine phosphate substrate per minute at 25°C and pH=6.8. In this study we expressed CK activity of seminal plasma as IU/L, sperm cells as IU / 108 sperm and total semen as IU/L.

### Statistical analysis

Results are presented as mean ± standard deviation (mean ± SD). All assays were performed in triplicate, and the mean was used for the calculation. Semen analysis and CK activity were compared using the independent samples t test in both smokers and nonsmokers. The t test was employed for comparisons between different subgroups. Coefficients of correlation were analyzed with linear (Pearson) analysis. P≤0.05 was considered statistically significant.

## Results

The study population consisted of 147 male participants. Smoking status was classified as follows: 56.5% (83/147) were nonsmokers, 29.2% (43/147) were short-term smokers, and 14.3% (21/147) were long-term smokers. There were 17.7% (26/147) light smokers, 20.4% (30/147) moderate smokers, and 5.4% (8/147) who were heavy smokers. Semen characteristics and CK activity in smokers and nonsmokers are given in table 3. Semen volume, concentration, motility, normal sperm morphology, and CK activity in semen, seminal plasma, and sperm in smokers were significantly lower than nonsmokers (Table 3).

There were statistically significant associations observed between sperm motility, CK activity in seminal plasma, sperm, and total semen in some of the subgroups of male smokers compared to nonsmokers (Table 4). There were no observed significant differences between sperm motility in short-term and light smokers compared to nonsmokers (Table 4).

**Fig 1:**
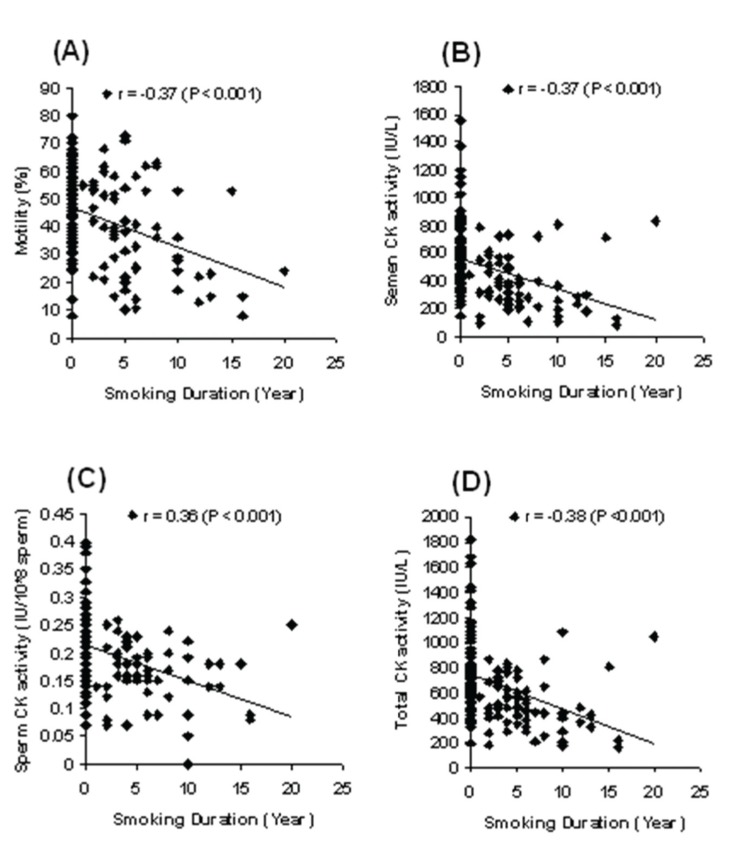
The relationship between smoking duration and sperm motility (A), CK activity in seminal plasma (B), sperm (C), and total semen (D).

**Fig 2:**
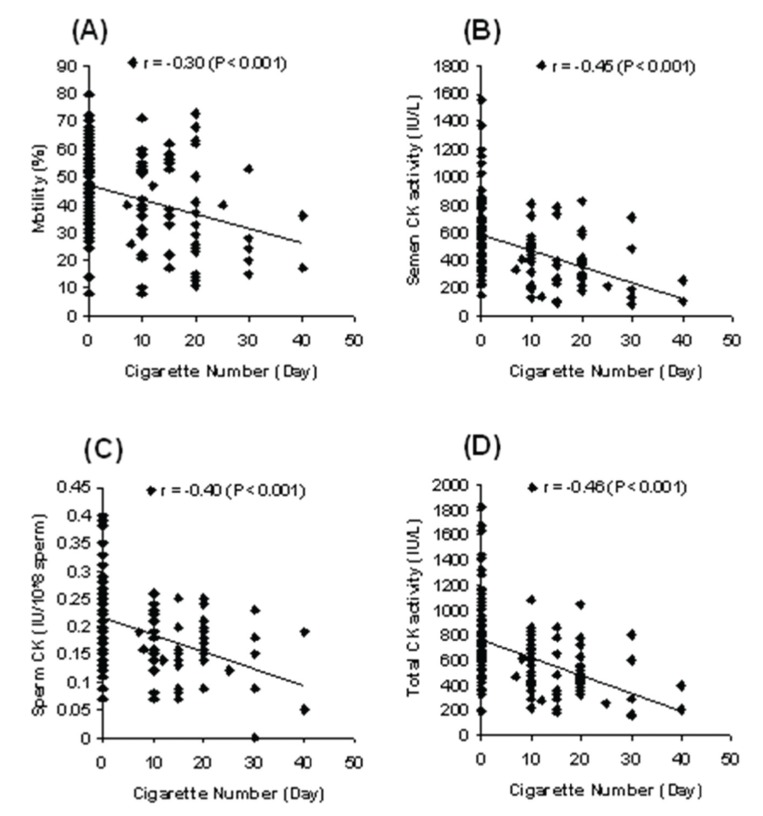
The relationship between numbers of cigarettes smoked daily and sperm motility (A), CK activity in seminal plasma (B), sperm (C), and total semen (D).

**Fig 3:**
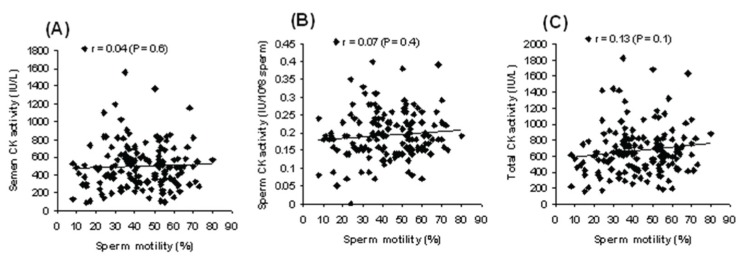
The relationship between sperm motility and CK activity in seminal plasma (A), sperm (B), and total semen (C) in smokers.

The same results were obtained for CK activity of sperm in short-term smokers (Table 4). The correlation between smoking duration, sperm motility, and CK activity of all analyzed samples is shown in figure 1. There were significant (p<0.001) negative correlations between smoking duration and sperm motility (r = -0.37), CK activity in seminal plasma (r = -0.37), sperm (r = -0.36), and total semen (r = -0.38). Figure 2 shows the correlation between the number of cigarettes smoked per day, sperm motility, and CK activity of all the analyzed samples. There were significant (p<0.001) negative correlations observed between the numbers of cigarettes smoked per day and sperm motility (r = -0.30), CK activity in seminal plasma (r = -0.45), sperm (r = -0.40), and total semen (r = -0.46). There were no significant positive correlations observed between sperm motility and CK activity of all the analyzed samples in smokers (Fig 3).

**Table 3:** Semen characteristics in non-smokers and smokers


Characteristics	Non-smokers (n = 83)	Smokers (n = 64)	P value

**Volume (ml)**	4.1 ± 1.4	3.2 ± 1.06	<0.001
**pH**	8.01 ± 0.22	8.03 ± 0.32	0.659
**Seminal plasma CK activity (IU/L)**	547.31 ± 193.8	377.83 ± 187.2	<0.001
**Total CK activity (IU/L)**	764.24 ± 259.7	510.03 ± 205.98	<0.001
**Sperm**			
**Concentration (10^6^ sperm/ml)**	65.5 ± 22.9	52.8 ± 20.7	0.001
**Motility (%)**	46.5 ± 13.8	38.6 ± 17.5	0.003
**Normal morphology (%)**	47.5 ± 18.9	37 ± 21.2	0.002
**CK activity (IU/10^8^ sperm)**	0.22 ± 0.07	0.16 ± 0.05	<0.001


**Table 4:** Sperm motility and CK activity of seminal plasma, sperm cells, total semen in smokers


Characteristics	Duration(Y)		Amount(cigarettes/day)			Non-smokers
	Short-term	Long-term	Light	Moderate	Heavy	

**Sperm motility (%)**	41.09 ± 18.4	31.9 ± 14.79*	41.38 ± 16.26	38.7 ± 19.02**	29.12 ± 13.05*	46.48 ± 13.84
**CK activity**	418.18 ± 198*	289.05 ± 189.99*	432.35 ± 33.02***	358.13 ± 185.51*	274.5 ± 216*	599.23 ± 246.89
**Seminal plasma (IU/L)**	0.2 ± 0.01	0.12 ± 0.05*	0.17 ± .04*	0.16 ± 0.05*	0.12 ± 0.05*	0.22 ± 0.07
**Sperm cells**	555.98 ± 186.4*	403.2 ± 214.4	577.27 ± 192*	490.57 ± 193.58*	356.12 ± 228.12*	788.48 ± 309.2
**Total semen (IU/L)**						


*;p<0.001, **; p=0.02 and***; p=0.002 vs. the corresponding values for non-smokers.

**Fig 4:**
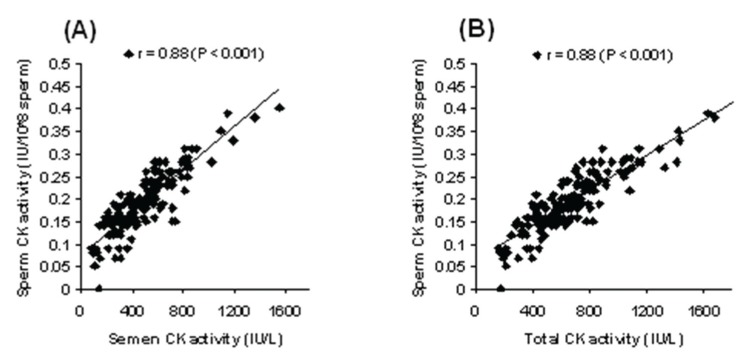
The relationship between sperm CK activity and CK activity in seminal plasma (A) and total semen (B) in smokers

There was a significant (r = 0.88, p<0.001) positive association among all the analyzed samples in smokers with respect to CK activity (Fig 4).

## Discussion

Smoking presents with a lifestyle hazard for those who smoke. Although the lungs are known to be a primary target for carcinogens in tobacco, numerous studies have suggested that smoking is associated with altered semen quality (5, 15). However, the effects of cigarette smoke on CK activity in human sperm cells, seminal plasma, and total semen is less documented. In the present study, we have observed a statistically significant relationship between cigarette smoking and several semen characteristics. Semen volume, sperm concentration, percentage of motile sperm and the percentage of normal morphology in sperm decreased with cigarette smoking. The findings of this study underlined the fact that smoking has an adverse influence on human semen quality as previously confirmed (2, 5, 15, 16). In addition, we have also shown that CK activity in seminal plasma, sperm cells and total semen significantly decreased with smoking.

This study demonstrated that smoking duration and number of cigarettes smoked per day affected sperm motility. Thus, we found that males who smoked for a duration of ≤10 years (short-term) had 12% decreased sperm motility whereas those who smoked for a duration of >10 and <20 years had 31% lower sperm motility than nonsmokers. Males who smoked ≤10 cigarettes per day (light) had an approximately 11% lower sperm motility, those who were moderate smokers (>10 and ≤20 cigarettes per day) had approximately 17% less motility and heavy (>20 cigarettes per day) smokers had approximately 37% lower sperm motility compared to nonsmokers. Pasqualotto et al. (17) reported a declining semen volume with an increased number of cigarettes smoked, but no significant differences were observed in sperm concentration, motility or morphology. An insignificant correlation was reported between sperm concentration and additional smoking (18).

The findings of the present study support those of numerous other studies, which show a significant relation between smoking duration, sperm concentration, and motility as well as between the number of cigarette smoked daily, sperm concentration and motility (5, 19-21). The mechanisms of effect of cigarette smoking on sperm quality, in particular sperm motility, are not fully understood. The nicotine and its metabolite for example, cotinine, are detectable in the seminal plasma of smokers. Therefore, it has been suggested that harmful components of tobacco smoke are able to pass through the blood-testis barrier (22). Zavos et al. (4) have reported reductions in sperm motility associated with abnormalities in the ultrastructure of the flagellum and the axonemal structures of the sperm tail. According to another report there was a negative correlation between sperm motility and the concentrations of cotinine and hydroxycotinine in seminal plasma (23). Ghaffari et al. (13) have suggested that some cigarette components such as nicotine, cotinine and cadmium can decrease human sperm CK activity in an *in vitro* model. The current investigation has shown that smoking duration and number of cigarettes smoked per day affect CK activity in seminal plasma, sperm cells, and total semen. According to these data, CK activity in seminal plasma (30%), sperm cells (9%), and total semen (29%) of males who smoked for a short time period were lower than in nonsmoking males. Additionally, CK activity in the seminal plasma (52%), sperm cells (45%) and total semen (49%) of long-term smokers were lower than male nonsmokers. We also observed that seminal plasma CK activity in light (28%), moderate (40%) and heavy (54%) smokers were lower than nonsmoking males as was sperm cell CK activity in light (23%), moderate (27%) and heavy (45%) smokers. Additionally, total semen CK activity also decreased in light (27%), moderate (38%), and heavy (55%) smokers. We could not locate any reports about the relationship between the number and duration of cigarette smoking with sperm cell and seminal plasma CK activity; reports essentially focused on CK activity in sperm cells and/or seminal plasma of normozoospermia, oligozoospermia and asthenozoospermia males (7, 24, 25).

CK has two distinct isomeric forms, brain CK (B-CK) and muscle CK (M-CK) which are present in the midpiece region and the sperm tail, respectively (26). Mature sperm show a greater concentration of the M-CK isoform, which is expressed only during the last phase of spermatogenesis in elongated spermatids and in mature sperm (27, 28). Huszar et al. (27) have demonstrated that M-CK concentration reflects sperm quality better than sperm concentration. Results of a study by Wallimann et al. (29) have shown that entails diffusion (What does this mean?) of phosphocreatine from the mitochondria to the axoneme and a countercurrent diffusion of creatine from the axoneme toward the mitochondria are the main factors for progressive motion in sperm cells.

According to Pearson's linear analysis, we found a significant negative relation between duration of cigarette smoking and sperm motility in smokers. A statistically significant negative correlation was also observed for CK activity in seminal plasma, sperm cells and total semen in smokers. In addition, our data showed that the daily number of cigarettes smoked had a significant negative effect on both sperm motility and CK activity in seminal plasma, sperm cells, and total semen.

The present study showed an insignificant positive relation between sperm motility and with CK activity of seminal plasma, sperm cells, and total semen samples in smokers. This investigation demonstrated a significant positive relationship between CK activity of sperm cells and activity of this enzyme in seminal plasma and total semen in smokers. The positive correlation between CK activity and sperm motility in smokers, has indicated that exposure to tobacco smoke can diminish sperm motility via inhibition of sperm CK activity. The insignificant correlation found in this study suggests the possibility of other causes that may be involved in this process.

These results show that maintaining of normal CK activity at physiological levels may provide an important contribution to sperm motility and fertility in males. According to previous research, it has been demonstrated that mammalian sperm must remain motile in the female tract and free energy released from the hydrolysis of ATP is required for this movement. Additionally, ADP can primarily be rephosphorylated by phosphocreatine. The rate of ATP synthesis via CK activity is generally faster than its rate of synthesis through oxidative phosphorylation (8).

## Conclusion

We found that cigarette smoking in adult males impaired sperm quality. This negative effect was dosedependent, as increased duration and quantity of cigarettes smoked had a positive effect on decreased sperm motility and CK activity. Therefore, we have suggested cigarette components that diminish sperm CK activity may potentially impair sperm energy homeostasis and thus have an association with damaged sperm motility. As a consequence, this effect can be one of the several important mechanisms that possibly cause infertility in male smokers. However, further research is required to elucidate the underlying mechanism of sperm motility damage caused by cigarette smoking.
